# Threshold-Based Hierarchical Clustering for Person Re-Identification

**DOI:** 10.3390/e23050522

**Published:** 2021-04-24

**Authors:** Minhui Hu, Kaiwei Zeng, Yaohua Wang, Yang Guo

**Affiliations:** College of Computer Science, National University of Defense Technology, Changsha 410073, China; huminhui09@nudt.edu.cn (M.H.); yhwang@nudt.edu.cn (Y.W.); guoyang@nudt.edu.cn (Y.G.)

**Keywords:** person re-identification, threshold-based hierarchical clustering, unsupervised domain adaptation, fully unsupervised method

## Abstract

Unsupervised domain adaptation is a challenging task in person re-identification (re-ID). Recently, cluster-based methods achieve good performance; clustering and training are two important phases in these methods. For clustering, one major issue of existing methods is that they do not fully exploit the information in outliers by either discarding outliers in clusters or simply merging outliers. For training, existing methods only use source features for pretraining and target features for fine-tuning and do not make full use of all valuable information in source datasets and target datasets. To solve these problems, we propose a **T**hreshold-based **H**ierarchical clustering method with **C**ontrastive loss (THC). There are two features of THC: (1) it regards outliers as single-sample clusters to participate in training. It well preserves the information in outliers without setting cluster number and combines advantages of existing clustering methods; (2) it uses contrastive loss to make full use of all valuable information, including source-class centroids, target-cluster centroids and single-sample clusters, thus achieving better performance. We conduct extensive experiments on Market-1501, DukeMTMC-reID and MSMT17. Results show our method achieves state of the art.

## 1. Introduction

Person re-identification(re-ID) is a practical task about finding a specific person from cameras and it is widely used in security fields. Unsupervised domain adaptation (UDA) re-ID [1,2,3] has been popular because it does not require a lot of labeled data with respect to supervised methods. UDA re-ID requires labeled source datasets and unlabeled target datasets. It usually conducts supervised learning on the source dataset to obtain a good pretraining model, then fine-tunes on the target dataset. Compared with supervised person re-ID algorithms [4,5], UDA re-ID reduces half the cost of manual annotation. More importantly, it achieves good performance on other datasets and is more suitable for practical application.

**Figure 1:**
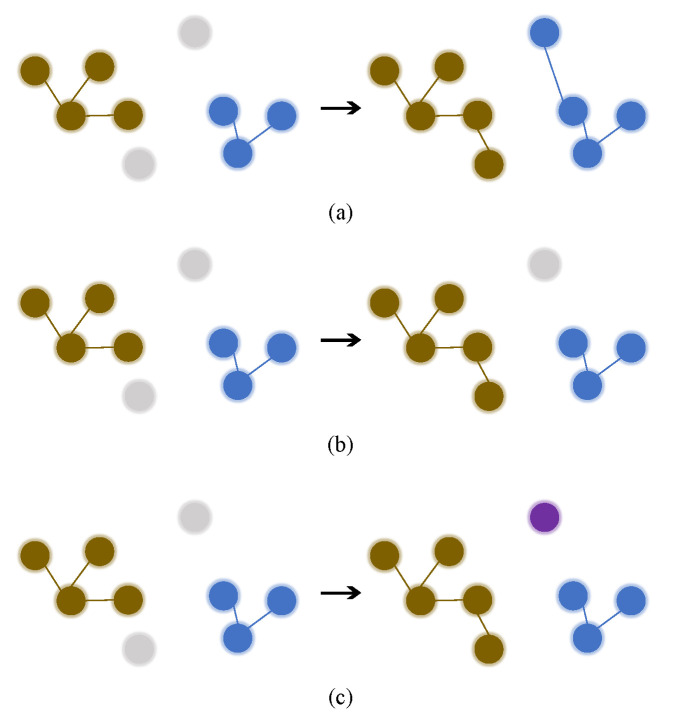
Comparison of three clustering methods. Each circle represents a sample, different colors mean different pesudo labels generated by clustering. The grey dot means outliers which will be discarded. (**a**) hierarchical clustering: outliers are forced to merge into the nearest clusters. (**b**) DBSCAN: if the distance between outliers and one sample in clusters is less than eps (threshold in DBSCAN, more details are in [10]), it will be clustered; otherwise it will be discarded. (**c**) threshold-based hierarchical clustering: if the distance between outliers and clusters is greater than threshold, it will be regarded as a single-sample cluster; otherwise it will be merged as usual.

Recent studies find that cluster-based methods achieve better performance. However, these methods do not deal with outliers well. As shown in Figure 1a, hierarchical clustering [6] merges the nearest two clusters in each step; even outliers will be forced to merge and generate noise labels for training. As shown in Figure 1b, DBSCAN [7] directly discards outliers; outliers will not participate in training. To tackle these problems, we propose a threshold-based hierarchical clustering method with contrastive loss (THC). As shown in Figure 1c, different from DBSCAN and hierarchical clustering, threshold-based hierarchical clustering regards outliers as single-sample clusters to generate pseudo labels and participate in training. It preserves all valuable information and does not need to set cluster number, so it combines advantages of DBSCAN and hierarchical clustering. We use a nonparametric feature memory [8] to store and update features. We use contrastive loss [9] to optimize model with source-class centroids, target-cluster centroids and single-sample clusters. Our method takes advantage of all valuable information and generates more reliable clusters, so we achieve better performance.

Our contributions can be summarized in three-fold:

- We propose a threshold-based hierarchical clustering method which combines advantages of hierarchical clustering and DBSCAN. It regards outliers as single-sample clusters to participate in training and generates more reliable pseudo labels for training.
- We propose to use nonparametric memory with contrastive loss to optimize model. We make full use of all valuable information including source-class centroids, target-cluster centroids and single-sample clusters, so we get better performance.
- We evaluate different distance measurements in threshold-based hierarchical clustering. Results show minimum distance criterion has the best performance. We also evaluate our method on three datasets: Market-1501, DukeMTMC-reID and MSMT17. Results show we achieve state of the art.

## 2. Related Work

### 2.1. Unsupervised Domain Adaptation re-ID

Although supervised person re-ID algorithms [4,5,11] have achieved good progress recently, they require a lot of annotated data and fail to transfer well to practical applications. Unsupervised Domain Adaptation re-ID (UDA re-ID) methods are promising to solve these problems. UDA person re-ID methods [1,6,7,12,13,14] can be divided into two categories: GAN-based methods and cluster-based methods. The former [12,15] focuses on differences between source domain and target domain. SPGAN [12] uses GAN [16] to translate image styles from source domain to target domain but keeps labels unchanged. It decreases differences between source domain and target domain and it augments data for training. However, images generated by these methods are obviously different from the real dataset, so the poor quality limits model performance. Some studies focus on auxiliary information. ECN [13] proposes three branches about exemplar-invariance [17,18], camera- invariance [19] and neighborhood-invariance [20]. ECN uses exemplar memory [21,22] to set pseudo labels for samples and optimizes the model with triplet loss. However, these methods introduce too much auxiliary information and interference.

Instead, cluster-based methods achieve better performance. PUL [1] uses *k*-means for clustering and only selects reliable samples for training according to the distance between samples and cluster centroids in each iteration. However, the clustering result of *k*-means is sensitive to outliers and cluster number, so PUL is unstable and has poor performance. BUC [6] proposes a bottom-up hierarchical clustering method to generate pseudo labels; it can better build the underlying structure of clusters by merging the most similar clusters step by step. However, the forced merging strategy generates noise labels for outliers and even leads to decline of model performance especially in the later merging stage. Besides, it is also difficult to set the cluster number in advance. Theory [7] proposes to combine DBSCAN with hard-batch triplet loss for fine-tuning. DBSCAN does not need to set the cluster number in advance and could automatically discard outliers during clustering, thus it achieves better performance. However, this simple discard strategy ignores the valuable information of outliers in the target dataset and limits model performance.

### 2.2. Noise Label Learning

Noise labels represent images which are difficult to be discriminated. However, these images also contain some important information. We cannot directly discard them. People focus on training with noise labels [23,24,25] in recent years. Based on coteaching [26], ACT [27] proposes a asymmetric coteaching structure and MMT [28] combines a mutual mean-teaching structure with soft pseudo labels to train outliers. These methods achieve some improvements, but they only use source features for pretraining and only use target features but ignore source features during fine-tuning. SPCL [29] regenerates pseudo labels for outliers in DBSCAN and trains model with all source features and target features. However, SPCL requires artificial definitions about compactness and independence degree. It also needs to redivide and regenerate pseudo labels for outliers to obtain more reliable clusters to further improve model performance.

### 2.3. Memory Module

Augmented-memory is widely used in question answering [30,31], few-shot learning [21] and video understanding [32]. It can be mainly divided into two categories: augmented neural networks [31] and nonparametric memory [8,17,18]. The latter is widely used in re-ID [13,29,33,34]. Nonparametric memory stores features in memory and update features through moving average during training. The memory module can fully exploit similarities between samples in the whole dataset instead of the mini-batch. It further improves model performance and only requires a little extra computation and GPU memory.

## 3. Our Method

**Figure 2:**
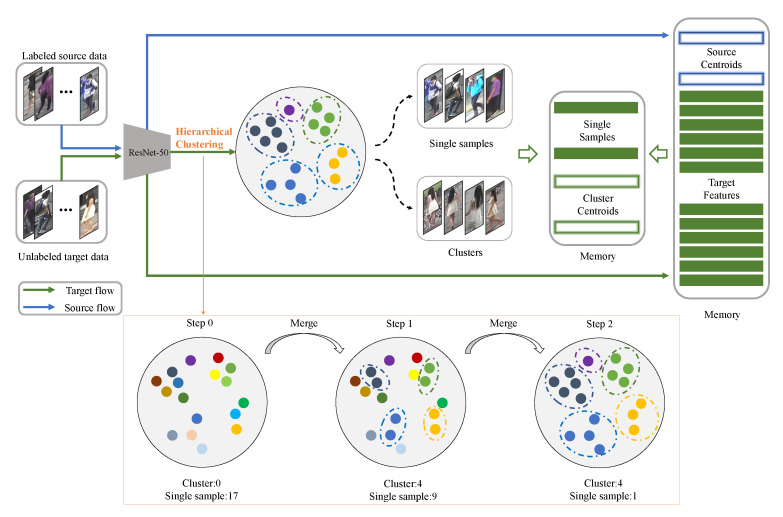
The framework of our method. During training, labeled source data and unlabeled target data participate in training together. We use threshold-based hierarchical clustering to divide clusters and single-sample clusters in the target dateset. We use pseudo labels generated by clustering to fine-tune the model with source-class centroids, target-cluster centroids and single-sample clusters and finally update features via memory.

Based on these studies, we propose a threshold-based hierarchical clustering method with contrastive loss. Our framework is shown in Figure 2. Specifically, (1) we use ResNet-50 [35] as backbone to extract features, then we use memory to store source-class centroids and target features, (2) we calculate distance between samples, update distance between clusters and generate pseudo labels by threshold-based hierarchical clustering, then we store target-cluster centroids and single-sample clusters features, (3) we use contrastive loss to optimize model and update memory features until we get the best performance.

### 3.1. Threshold-Based Hierarchical Clustering

#### 3.1.1. Distance Metric

Given a target dataset $X_{T}=\left\{x_{1}^{t},x_{2}^{t},\cdots ,x_{n_{t}}^{t}\right\}$, we use *k*-reciprocal encoding [36] to calculate the distance:

(1) $$M_{ij}=\left\{\begin{array}{c} e^{-{∥x_{i}^{t}-x_{j}^{t}∥}^{2}},j\in R^{*}\left(i,k\right)\\ 0,\;otherwise\; \end{array}\right.$$

where ${∥\cdot ∥}^{2}$ represents the euclidean distance, $R^{*}\left(i,k\right)$ is the *k*-reciprocal set for sample $x_{i}^{t}$, *M* is a $n_{t}\times n_{t}$ metric, $n_{t}$ is the sample number in the target dataset. Finally, we use the Jaccard distance as the final distance between samples for clustering:

(2) $$d_{J}\left(x_{i}^{t},x_{j}^{t}\right)=1-\frac{\sum_{k=1}^{n}\min\left(M_{ik},M_{jk}\right)}{\sum_{k=1}^{n}\max\left(M_{ik},M_{jk}\right)}$$

#### 3.1.2. Hierarchical Cluster Merging

As depicted in Figure 2, in the beginning of hierarchical clustering, we regard samples as single-sample clusters and generate different pseudo labels for them. It merges the nearest two clusters in each step from bottom to up and updates labels. However, this original strategy forced gradually merging outliers into clusters and generates lots of noise labels, especially in the later merging stage. It also needs to set the cluster number in advance like *k*-means. The clustering result is sensitive to cluster number and finally limits model performance.

To tackle these problems, we set a hyperparameter threshold for hierarchical clustering. During clustering, two clusters will be merged only if the distance between them is less than threshold. We conducted experiments in Section 5.1 to define the value of threshold. This strategy has two advantages. (1) Outliers will not be directly discarded or forced to merge into the nearest cluster. If the distance is greater than threshold. they will be regarded as single-sample clusters to participate in training. (2) Similar to DBSCAN, we do not need to set the cluster number in advance; it can make the model more stable. So it can obtain more reliable clustering results to improve model performance.

#### 3.1.3. Distance Measurement

Distance measurement is important in hierarchical clustering because it decides which two clusters will be merged. We choose the minimum distance criterion in our experiments. It only considers the shortest distance between images in two clusters. If these two images are similar, two clusters will be merged no matter how dissimilar other images are. We argue that images of the same identity under the same camera are prior to be merged under this criterion. The formula is:

(3) $$D\left(A,B\right)=\min_{x_{a}\in A,x_{b}\in B}d_{J}\left(x_{a},x_{b}\right)$$

where D(A,B) means the distance between cluster *A* and cluster *B*. We also discuss other criterions. (1) The maximum distance criterion only considers the maximum distance between images in two clusters. The formula is:

(4) $$D\left(A,B\right)=\max_{x_{a}\in A,x_{b}\in B}d_{J}\left(x_{a},x_{b}\right)$$

(2) The average distance criterion considers all pairwise distance between images in two clusters and each distance has the same weight. The formula is:

(5) $$D\left(A,B\right)=\frac{1}{n_{a}n_{b}}\sum_{x_{a}\in A,x_{b}\in B}d_{J}\left(x_{a},x_{b}\right)$$

where $n_{a},n_{b}$ is the image number in cluster *A* and cluster *B*. We discuss performance of different criterions in Section 5.1 and demonstrate the minimum distance criterion get the best performance.

### 3.2. Nonparametric Memory

#### 3.2.1. Memory Initialization

As shown in Figure 2, given a source dataset $X_{S}=\left\{x_{1}^{s},x_{2}^{s},\cdots ,x_{n_{s}}^{s}\right\}$, we extract features: $S=\left\{s_{1},s_{2},\cdots ,s_{n_{s}}\right\}$. We calculate the mean of all features in each class and store source-class centroids features $\left\{f_{1},f_{2},\cdots ,f_{n_{c}^{s}}\right\}$:

(6) $$f_{i}=\frac{1}{\left|C_{i}^{s}\right|}\sum_{s_{i}\in C_{i}^{s}}s_{i}$$

where $f_{i}$ is the source-class centroid of the *i*-th class $C_{i}^{s}$, $\left|\cdot \right|$ is the image number in the class, $n_{c}^{s}$ is the class number in the source dataset, $n_{s}$ is the sample number in the source dataset.

Given a target dataset $X_{T}=\left\{x_{1}^{t},x_{2}^{t},\cdots ,x_{n_{t}}^{t}\right\}$, we store all target features: $T=\left\{t_{1},t_{2},\cdots ,t_{n_{t}}\right\}$. After clustering, we calculate the mean of all features in each cluster and store target-cluster centroids features $\left\{c_{1},c_{2},\cdots ,c_{n_{c}^{t}}\right\}$:

(7) $$c_{i}=\frac{1}{\left|C_{i}^{t}\right|}\sum_{t_{i}\in C_{i}^{t}}t_{i}$$

where $c_{i}$ is the target-cluster centroid of the *i*-th cluster $C_{i}^{t}$, $n_{c}^{t}$ is the cluster number in the target dataset, $n_{t}$ is the sample number in the target dataset. For single-sample clusters, we directly copy features in T and store them as $\left\{v_{1},v_{2},\cdots ,v_{n_{s}^{t}}\right\}$, where $n_{s}^{t}$ is the number of single-sample clusters in the target dataset.

#### 3.2.2. Memory Update

**Figure 3:**
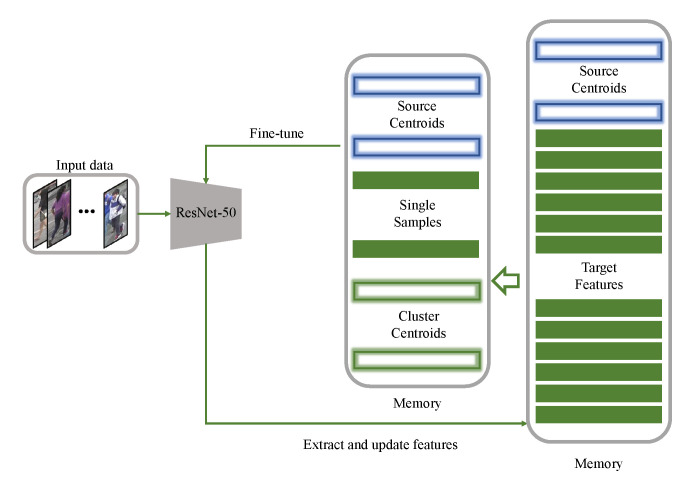
The framework of memory update and model fine-tuning. We use Resnet-50 to extract features to initialize memory. Subsequently, we fine-tune model and update features via memory by moving average.

As shown in Figure 3, we use ResNet-50 to extract features and initialize memory in the beginning. In subsequent iterations, we fine-tune model with memory features and use moving average to update memory features. For source-class centroids:

(8) $$f_{i}\leftarrow \alpha_{s}f_{i}+\left(1-\alpha_{s}\right)\cdot \frac{1}{\left|C_{i}^{s}\right|}\sum_{s_{i}\in C_{i}^{s}}s_{i}$$

where $\alpha_{s}$ is the update rate of $f_{i}$, we empirically set it to 0.2. For target features:

(9) $$t_{i}\leftarrow \alpha_{t}t_{i}+\left(1-\alpha_{t}\right)\cdot f\left(x_{i}^{t}\right)$$

where $\alpha_{t}$ is the update rate of $t_{i}$, we empirically set it to 0.2, $f\left(\cdot \right)$ is the encoder, $x_{i}^{t}$ is the sample in the target dataset. If $t_{i}$ belongs to cluster $C_{i}^{t}$, we calculate the *i*-th target-cluster centroid features $c_{i}$ through Equation (7). Finally, we update $\left\{c_{i}\right\}$ and single-sample clusters features $\left\{v_{i}\right\}$ in memory for training. The detail is shown in Algorithm 1.
**Algorithm 1** THC Algorithm**Require:**
       Labeled source dataset $X_{S}$;

       Unlabeled target dataset $X_{T}$;

       Epoch *t*;

       Threshold *m*;

       Update rate $\alpha_{s}$,$\alpha_{t}$.
**Ensure:**       Best model *M*.
1:Extract all source features S and target features T;2:Calculate source-class centroids according to Equation (6), store source-class centroids $\left\{f_{i}\right\}$ and target features T in memory;3:**for**i=0 to *t* **do**4:    Obtain target features T from memory, calculate Jaccard distance between samples according to Equations (1) and (2);5:    Use threshold-based hierarchical clustering to cluster samples according to Equation (3), generate pseudo labels based on clustering results;6:    Calculate target-cluster centroids according to Equation (7), store target-cluster centroids $\left\{c_{i}\right\}$ and single-sample clusters $\left\{v_{i}\right\}$ in memory;7:    Fine-tune model with contrastive loss and evaluate model performance;8:    **if** $mAP_{i}>mAP_{best}$ **then**9:         $mAP_{best}=mAP_{i}$,         update best model *M*;10:    **end if**11:    Update target features T, calculate and update source-class centroids $\left\{f_{i}\right\}$, target-cluster centroids $\left\{c_{i}\right\}$ and single-sample clusters $\left\{v_{i}\right\}$ according to Equations (6)–(9);12:**end for**

### 3.3. Loss Function

We use contrastive loss to optimize model; the loss is defined as:

(10) $$L=-\log\frac{\exp\left(\langle f\left(x_{j}\right),p^{+}\rangle /\tau \right)}{\sum_{i=1}^{n_{c}^{s}}\exp\left(\langle f\left(x_{j}\right),f_{i}\rangle /\tau \right)+\sum_{i=1}^{n_{c}^{t}}\exp\left(\langle f\left(x_{j}\right),c_{i}\rangle /\tau \right)+\sum_{i=1}^{n_{s}^{t}}\exp\left(\langle f\left(x_{j}\right),v_{i}\rangle /\tau \right)}$$

where $\langle \cdot \rangle$ is the inner product to measure the similarity between two samples, τ is a temperature parameter, we empirically set it to 0.05. Note that $x_{j}$ is a sample from the source dataset or target dataset, $p^{+}$ is the positive class about $f\left(x_{j}\right)$. If $x_{j}$ is a sample in the source dataset, $p^{+}=f_{i}$ is the source-class centroid that $x_{j}$ belongs to. If $x_{j}$ is a sample in clusters, $p^{+}=c_{i}$ is the target-cluster centroid that $x_{j}$ belongs to. If $x_{j}$ is a single-sample cluster, $p^{+}=v_{i}$ is the feature vector about $x_{j}$. Through contrastive loss, we make fully use of all valuable information about source-class centroids, target-cluster centroids and single-sample clusters, so we get better performance. We can also further reduce the need of source labeled datasets, then contrastive loss is defined as:

(11) $$L=-\log\frac{\exp\left(\langle f\left(x_{j}\right),p^{+}\rangle /\tau \right)}{\sum_{i=1}^{n_{c}^{t}}\exp\left(\langle f\left(x_{j}\right),c_{i}\rangle /\tau \right)+\sum_{i=1}^{n_{s}^{t}}\exp\left(\langle f\left(x_{j}\right),v_{i}\rangle /\tau \right)}$$

We discuss model performance about this fully unsupervised setting in Section 4.4.

## 4. Experiment

### 4.1. Datasets

**Market1501** Market-1501 [37] includes 32,668 images of 1501 pedestrians captured by 6 cameras. There are 751 identities of 12,936 images in the training set and 750 identities of 19,732 images in the test set.

**DukeMTMC-reID** DukeMTMC-reID [38] is a subset of DukeMTMC [39]. DukeMTMC contains 85 minutes high-resolution video from eight cameras. DukeMTMC-reID contains 16,522 images about 702 identities for training, 2228 query images about the other 702 identities and 17,661 gallery images for testing.

**MSMT17** MSMT17 [15] contains 126,441 boxes of 4101 identities taken by 12 outdoor cameras and 3 indoor cameras in four days. The training set includes 1041 identities with 32,621 bounding boxes, the test set includes 3060 identities with 93,820 bounding boxes. For the test set, 11,659 bounding boxes are randomly selected as query and the other 82,161 bounding boxes are gallery.

### 4.2. Evaluation Protocol

In our experiment, we use the single-query setting to evaluate model performance. We take mean average precision (mAP) and rank-n scores as performance indicators based on cumulative matching characteristic (CMC). Importantly, we do not adopt postprocessing methods for testing such as reranking [36] or multiquery [37]. We evaluate our model on four RTX 2080 based on CUDA 10.0 and Pytorch 1.0.

### 4.3. Implementation Details

**Data Processing** We resize images to 256 × 128 and adopt random cropping, flipping, random erasing [40] for data augmentation. For the source dataset, we randomly select 4 images from each of 16 identities (mini-batch =4×16=64). For the target dataset, we randomly select 64 images of at least 16 identities (4 images for each cluster or 1 image for each single-sample cluster).

**Training Setting** We use pretrained ResNet-50 [35] on ImageNet as the backbone. We use domain-specific BNs [41] to narrow domain gaps. We use Adam [42] to optimize model with decay of 0.0005. The total epoch is 50 and each epoch has 400 iterations. The learning rate is 0.00035 and decreased by 1/10 every 20 epochs. The temperature τ is 0.05, update rate $\alpha_{s}=\alpha_{t}=0.2$. For clustering, we use the minimum distance criterion, threshold is set to 0.51 for Market-1501 and DukeMTMC-reID, 0.58 for MSMT17.

### 4.4. Comparison with State-of-the-Arts

**Comparison with UDA methods** We compare our methods with other UDA methods. As shown in Table 1. We achieve rank-1 = 91.2%, mAP = 78.5% on DukeMTMC-reID → Market-1501 and rank-1 = 83.0%, mAP = 68.8% on Market-1501 → DukeMTMC-reID. We surpasses other state-of-the-art methods. More importantly, we do not need to set cluster number for target datasets like MMT [28] and MEB-Net [43]. Unsupervised re-ID is an open task in which it is difficult to know cluster number in advance, so our method is more applicable in real life.

We also evaluate our method on a challenging dataset MSMT17, as shown in Table 2. We achieve mAP = 23.7% on Market-1501 → MSMT17 and mAP = 24.9% on DukeMTMC-reID → MSMT17. Our method has better performance on mAP compared with other methods, which further demonstrates the validation of our method.

**Comparison with fully unsupervised methods.** We adopt the fully unsupervised setting described in Section 3.3. Results are reported in Table 3. We achieve rank-1 = 89.5%, mAP = 75.2% on Market-1501 and rank-1 = 81.9%, mAP = 66.2% on DukeMTMC-reID. Compared with state-of-the-art method HCT [50], we achieve rank-1 = 9.5%, mAP = 18.8% promotion on Market-1501 and rank-1 = 12.3%, mAP = 15.5% promotion on DukeMTMC-reID. Results prove our model has better performance on both UDA and fully unsupervised task.

## 5. Ablation Study

### 5.1. Comparison with Different Distance Measurements and Threshold Values

**Figure 4:**
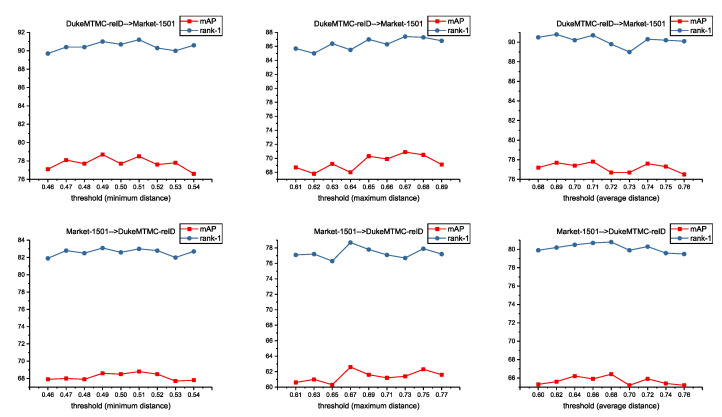
Comparison with different distance measurements and threshold values on Market-1501 and DukeMTMC-reID.

Threshold-based hierarchical clustering merges clusters from the bottom up, step by step. The clustering result highly depends on the distance measurement and the value of threshold. In our experiments, we compare minimum distance criterion, maximum distance criterion and average distance criterion. For each criterion, we set different threshold values to find the best performance. Results are shown in Figure 4. We get best performance on DukeMTMC-reID → Market-1501 and Market-1501 → DukeMTMC-reID with minimum distance criterion. There is only a little performance difference between the minimum distance criterion and average distance criterion but a huge difference compared with maximum distance criterion.

### 5.2. Comparison with Clusters Number during Training

**Figure 5:**
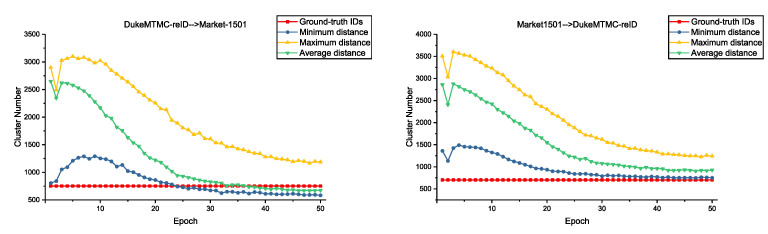
Change of clusters’ number during training under different distance measurements on Market-1501 and DukeMTMC-reID.

In order to better reflect the difference of different distance measurements, we record the change of clusters number in each epoch on DukeMTMC-reID → Market-1501 and Market-1501 → DukeMTMC-reID. Results are shown in Figure 5. We find that clusters’ number has the smallest change and the curve is close to the real number under the minimum distance. Instead, cluster number has the largest change and the curve is hard to get close to the real number under the maximum distance. We argue images of the same identity under the same camera are similar; they are easy to be merged with minimum distance. However, images of the same identity under different cameras are dissimilar; it is difficult for them to be merged with maximum distance. It finally leads to too many clusters and poor clustering results. Average distance considers all pairwise distance, so its performance is between them.

### 5.3. Qualitative Analysis of T-SNE Visualization

**Figure 6:**
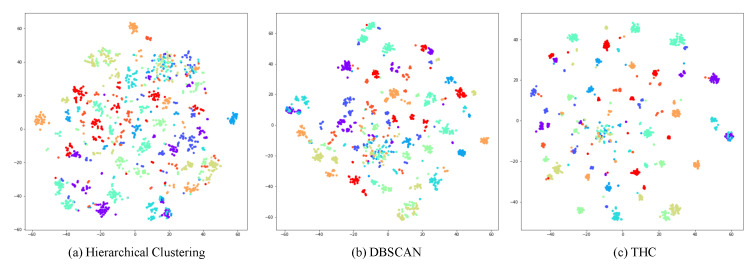
T-SNE visualization of different clustering methods. We choose a subset about 100 identities of Market-1501. Different color means different real labels. Those samples close to each other have the same pseudo label in clustering.

As shown in Figure 6, compared to a hierarchical clustering method BUC [6] and a DBSCAN method theory [7], our method can promote more compact clusters. Hierarchical clustering forces outliers to merge, DBSCAN directly discards them and threshold-based hierarchical clustering regards them as single-sample clusters to participate in training. We argue it can further improve model performance by discriminating outliers better than other methods.

## 6. Discussion

Inspired by previous critical work about hierarchical models [52,53,54,55,56] in various computer vision tasks, we propose our threshold-based hierarchical clustering method for re-ID. We follow the core idea of bottom-up hierarchical method to get reliable results for clustering target features. We utilize outliers as supervisions instead of discarding them directly as conventional methods [17,50]. We also set a hyperparameter threshold to prevent forcing merging outliers in hierarchical clustering. As a result, we get compatible performance with state-of-the-arts. However, we also find our performance on rank-*k* is slightly poorer than MMT [28] in Table 2. We believe threshold prevents the merging of clusters. This strategy promotes the whole quality of clusters, but it also ignores some similar samples and finally results in lower rank-*k* and higher mAP.

As shown in Table 1, our method achieves rank-1 = 91.2%, mAP = 78.5% on DukeMTMC-reID → Market-1501 and rank-1 = 83.0%, mAP=68.8% on Market-1501 → DukeMTMC-reID. However, when we apply our method on Market-1501 → MSMT17, there is a huge decline of performance with rank-1 = 48.2% and mAP = 23.7%. This phenomenon also appears in other advanced methods as shown in Table 2. The reason of this phenomenon may be that MSMT17 is a much bigger dataset than Market1501 and DukeMTMC-reID. After the model is trained on Market-1501, the model may have a certain degree of overfitting. As a result, the performance of re-ID models decline a lot when transferred to MSMT17. Although annotating person re-ID dataset is costly and time-consuming, larger datasets of person re-ID are needed to make person re-ID models work effectively in the real world.

## 7. Conclusions and Future Work

In this paper, we propose a threshold-based hierarchical clustering method for re-ID. Threshold-based hierarchical clustering regards outliers as single-sample clusters to participate in training with source-class centroids and target-cluster centroids through contrastive loss. The proposed method performs well on three large scale datasets in both unsupervised domain adaptation and fully unsupervised task. We hope our method can provide an option for future application of person reidentification.

In our threshold-based hierarchical clustering method, we use the original DBSCAN algorithm for clustering. Our future work intended to optimize this inner clustering method. In DBSCAN, we use the mean to calculate the centroids of source domain classes and target domain clusters. Since using the median may lead to more robust clustering results [57], further research would be done to explore the influence of the median. We also intend to optimize the distance metric and choice of core point in DBSCAN.

## Figures and Tables

**Table 1:** Comparison with state-of-the-art methods on Market-1501 and DukeMTMC-reID. Results that surpass all methods are **bold**.

Methods	DukeMTMC-reID → Market-1501				Market-1501 → DukeMTMC-reID			
	Rank-1	Rank-5	Rank-10	mAP	Rank-1	Rank-5	Rank-10	mAP
PUL [1]	44.7	59.1	65.6	20.1	30.4	44.5	50.7	16.4
SPGAN [12]	51.5	70.1	76.8	22.8	41.1	56.6	63.0	22.3
HHL [19]	62.2	78.8	84.0	31.4	46.9	61.0	66.7	27.2
ARN [44]	70.3	80.4	86.3	39.4	60.2	73.9	79.5	33.4
MAR [45]	67.7	81.9	-	40.0	67.1	79.8	-	48.0
ECN [13]	75.1	87.6	91.6	43.0	63.3	75.8	80.4	40.4
EANet [46]	78.0	-	-	51.6	67.7	-	-	48.0
Theory [7]	75.8	89.5	93.2	53.7	68.4	80.1	83.5	49.0
PAST [47]	78.4	-	-	54.6	72.4	-	-	54.3
SSG [14]	80.0	90.0	92.4	58.3	73.0	80.6	83.2	53.4
MMCL [48]	84.4	92.8	95.0	60.4	72.4	82.9	85.0	51.4
ACT [27]	80.5	-	-	60.6	72.4	-	-	54.5
ECN++ [33]	84.1	92.8	95.4	63.8	74.0	83.7	87.4	54.4
DG-Net++ [49]	82.1	90.2	92.7	61.7	78.9	87.8	90.4	63.8
MMT [28]	87.7	94.9	96.9	71.2	78.0	88.8	92.5	65.1
MEB-Net [43]	89.9	96.0	97.5	76.0	79.6	88.3	92.2	66.1
THC	**91.2**	**96.4**	**97.7**	**78.5**	**83.0**	**90.1**	**92.7**	**68.8**

**Table 2:** Comparison with state-of-the-art methods on MSMT-17.

Methods	Market-1501 → MSMT17				DukeMTMC-reID → MSMT17			
	Rank-1	Rank-5	Rank-10	mAP	Rank-1	Rank-5	Rank-10	mAP
PTGAN [15]	10.2	-	24.4	2.9	11.8	-	27.4	3.3
ECN [13]	25.3	36.3	42.1	8.5	30.2	41.5	46.8	10.2
SSG [14]	31.6	-	49.6	13.2	32.2	-	51.2	13.3
MMCL [48]	40.8	51.8	56.7	15.1	43.6	54.3	58.9	16.2
ECN++ [33]	40.4	53.1	58.7	15.2	42.5	55.9	61.5	16.0
DG-Net++ [49]	48.4	60.9	66.1	22.1	48.8	60.9	65.9	22.1
MMT [28]	**49.2**	**63.1**	**68.8**	22.9	**50.1**	**63.9**	**69.8**	23.3
THC	48.2	59.7	64.5	**23.7**	50.0	61.5	66.7	**24.9**

**Table 3:** Comparison with state-of-the-art fully unsupervised methods only with unlabeled target datasets on Market-1501 and DukeMTMC-reID.

Methods	Market-1501				DukeMTMC-reID			
	Rank-1	Rank-5	Rank-10	mAP	Rank-1	Rank-5	Rank-10	mAP
BOW [37]	35.8	52.4	60.3	14.8	17.1	28.8	34.9	8.3
OIM [18]	38.0	58.0	66.3	14.0	24.5	38.8	46.0	11.3
BUC [6]	66.2	79.6	84.5	38.3	47.4	62.6	68.4	27.5
SSL [51]	71.7	83.8	87.4	37.8	52.5	63.5	68.9	28.6
MMCL [48]	80.3	89.4	92.3	45.4	65.2	75.9	80.0	40.9
HCT [50]	80.0	91.6	95.2	56.4	69.6	83.4	87.4	50.7
THC	**89.5**	**95.8**	**97.5**	**75.2**	**81.9**	**89.9**	**92.5**	**66.2**

## Data Availability

Publicly available datasets were analyzed in this study. This three datasets used in this study can be found here: https://www.kaggle.com/pengcw1/market-1501/data; https://exposing.ai/duke_mtmc/; http://www.pkuvmc.com, accessed on 23 April 2021.
